# Food insecurity and patterns of dietary intake in a sample of UK adults

**DOI:** 10.1017/S0007114521003810

**Published:** 2022-08-28

**Authors:** Jackie Shinwell, Melissa Bateson, Daniel Nettle, Gillian V. Pepper

**Affiliations:** 1 Department of Social Work, Education and Community Wellbeing, Northumbria University, Newcastle, UK; 2 Biosciences Institute, Newcastle University, Newcastle, UK; 3 Population Health Sciences Institute, Newcastle University, Newcastle, UK; 4 Department of Psychology, Northumbria University, Newcastle, UK

**Keywords:** Dietary intake, Food insecurity, Meal timings, US, UK

## Abstract

The aim of this study was to identify the dietary intake correlates of food insecurity (FI) in UK adults. We recruited groups of low-income participants who were classified as food insecure (*n* 196) or food secure (*n* 198). Participants completed up to five 24 h dietary recalls. There was no difference in total energy intake by FI status (*β*_FI_ = −0·06, 95 % CI − 0·25, 0·13). Food insecure participants consumed a less diverse diet, as evidenced by fewer distinct foods per meal (*β*_FI_ = −0·27, 95 % CI − 0·47, −0·07), and had more variable time gaps between meals (*β*_FI_ = 0·21, 95 % CI 0·01, 0·41). These associations corresponded closely to those found in a recent US study using similar measures, suggesting that the dietary intake signature of FI generalises across populations. The findings suggest that the consequences of FI for weight gain and health are not due to increased energy intake. We suggest that there may be important health and metabolic effects of temporal irregularity in dietary intake, which appears to be an important component of FI.

Food insecurity (FI) is defined as the ‘the inability to acquire or consume an adequate quality or sufficient quantity of food in socially acceptable ways, or the uncertainty that one will be able to do so^(1)^. FI is associated with poorer health, higher mortality and, in the case of women, a greater risk of overweight or obesity, even after adjusting for socio-economic position^(2–8)^. Some of these health consequences may be due to different patterns of dietary intake in people experiencing FI compared with people who are not^(9–11)^. However, understanding is currently limited of how patterns of dietary intake vary with FI status. The questionnaires used to assess FI consist of general statements such as ‘we couldn’t afford to eat balanced meals’. Hence, although positive questionnaire responses suggest altered dietary intake, they are uninformative as to exactly how it differs. Datasets are therefore required where the same individuals complete FI questionnaires and separately provide detailed dietary recall information.

Where such studies have been carried out, people experiencing FI have not been found to have higher total energy intake^(9,12–15)^. Their intake has sometimes been found to differ in other ways. Overall measures of dietary quality are poorer in some but not all studies(16). Several studies have found reduced consumption of fruit and vegetables, and consequently fibre, associated with FI, though again findings are mixed^(16)^. Nettle and Bateson^(10)^ used data from the US National Health and Nutrition Examination Survey (NHANES) 2013–2014 to study a range of dietary intake parameters in relation to FI status. They confirmed that there was no difference in total energy intake. The largest differences they found were that people experiencing FI had more variable time gaps between eating and had a less diverse intake (fewer distinct foods per meal). These differences in eating patterns partially mediated the association (in women) between FI status and BMI. These findings have not yet been replicated in any other population.

The goal of the present study was to examine how dietary intake patterns differed by FI status in a UK population. In a two-stage design, we first targeted a sector of the population (adults from households below the UK median income) likely to contain substantial exposure to FI. We then invited all individuals classified by questionnaire as food insecure, and an equal number of demographically similar food secure individuals, to complete four, 24 h dietary recalls. We measured the same dietary intake parameters as Nettle and Bateson^(10)^ had done for the US NHANES sample. Our aims were: first, to examine which dietary intake parameters differed by FI status, and how, in this UK sample; and second, to directly compare the associations between FI status and dietary intake across the UK sample and the NHANES 2013–2014 sample studied by^(10)^. To facilitate direct comparison, we represent the key analyses of the NHANES 2013–2014 data in this paper, alongside the UK ones.

## Materials and methods

**Fig. 1.**
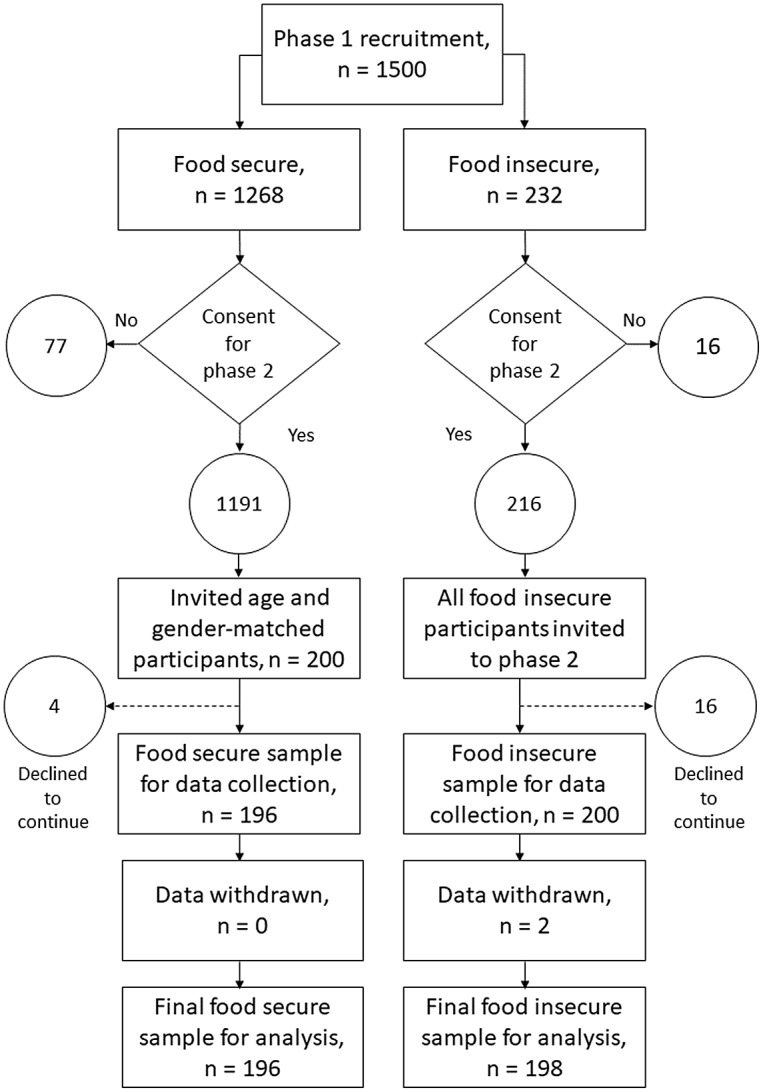
Flow chart depicting the recruitment and sampling (phase 1) and data collection (phase 2) stages of the study.

Data for the present UK study were collected in two phases, as depicted in Fig. 1. Phase 1 of the study was a screening exercise used to identify potential participants for phase 2.

### Phase 1

#### Participant recruitment

Participants were recruited to phase 1 of this study via Prolific, an internet-based crowd sourcing platform that provides access to a high quality pool of potential research participants (for a review of Prolific, see^(17)^). All participants were resident in the UK, were not in full-time education and had a total household income of less than £30 800 per annum (the median household income in the UK^(18)^). The household income filter was applied in order to identify individuals likely to experience FI, as well as food secure participants of similar socio-economic status. A total of 1500 participants were recruited to phase 1 of the study (male, 598, female, 898, other, 4, see online Supplementary Table S1).

#### Demographic data

Demographic data were collected via a Qualtrics questionnaire distributed via Prolific (see online Supplementary Table S1). Measures included age, sex, self-reported weight and height (from which we calculated BMI), number of people living in the household, whether participants did/paid for most of the food shopping for the household, employment status and household income. Household income was assessed by first presenting a choice of weekly, monthly or annual reporting, and then 24 income bands. These were taken from the Food and You survey, wave 5^(19)^. Income band was used in statistical models but we converted to pounds, using the mid-point of the band, for Table 1.

**Table 1.** Demographic profile of final UK sample (Data are frequencies or means and standard deviation)

Characteristic	Food secure			Food insecure			Difference
	Frequencies	Mean	sd	Frequencies	Mean	sd	
*n*	196			198			
Age		35·80	10·99		35·53	10·71	*t* = 0·25, *P* = 0·80
Income		17 840	8213		16 008	9316	*t* = 2·07, *P* = 0·04
Male	82			87			χ^2^ = 0·08, *P* = 0·78
Employed	143			124			χ^2^ = 4·69, *P* = 0·03
Unemployed/furloughed/Retired/ill health, etc.	52			74			χ^2^ = 4·69, *P* = 0·03
Household type							χ^2^ = 7·87, *P* = 0·02
Homeowner	61			39			
Rent	90			115			
Live with parents	44			44			
Number of people living in the household		2·98	1·22		2·88	1·38	*t* = 0·76, *P* = 0·44

#### Food insecurity

Household FI status was assessed using the Household Food Security Survey Six-Item Short Form module developed by the US National Centre for Health Statistics^(20)^. The six-item short form module identifies households which are food insecure and/or have very low food security. It has relatively high specificity compared with the longer, 18 item measure developed by the National Centre for Health Statistics^(20)^.

Data for this study were collected during spring/summer 2020, during which pandemic lockdowns had caused turmoil and disruption to normal routines. The type of FI experience we were interested in was that directly comparable to earlier studies, not shorter-term anxieties that might have been provoked by the onset of the pandemic. We thus amended the Household Food Security Survey Six-Item Short Form module questions from their usual ‘in the last 12 months’ timeframe to ‘in the 12-month period prior to the pandemic’.

Responses to questions in the Household Food Security Survey Six-Item Short Form module were coded in accordance with guidance issued by the USA National Centre for Health Research^(20)^. In accordance with USDA Household Food Security Survey Six-Item Short Form module guidance, we dichotomised participants’ scores into two groups, food secure/marginal food security (score of 0–1) (*n* 1268) and low/very low food security (score of 2–6) (*n* 232).

### Phase 2

#### Participant recruitment

Data collected in phase 1 were used to identify participants for phase 2 of the study (see Fig. 1). At the end of phase 1, participants were asked to indicate if they could be contacted about participating in phase 2 of the study. A total of ninety-three participants (seventy-seven high or marginal food security status, sixteen low or very low food security status) indicated that they did not want to take part in phase 2, resulting in a potential phase 2 participant pool of 1407. We invited 216 participants who had low/very low food security status to participate in phase 2 of the study. Sixteen participants declined to take part. The remaining 200 low/very low food security status participants were then matched by age and sex with 200 participants who had high/marginal food security status in phase 1. Four food secure participants declined to take part.

In addition, in accordance with guidance from the developers of Intake24, the dietary intake collection tool (see below), dietary intake data on two low/very low food secure participants were excluded from the analysis due to incorrect food diary completion. This resulted in a final sample for analysis of 198 low/very low food secure participants and 196 high/marginal food secure participants. For brevity, we henceforth refer to these groups as ‘food insecure’ and ‘food secure’, respectively. This final sample size, which was determined by logistical constraints, provided a minimum detectable difference of *d* = 0·28 between food insecure and food secure groups, with *P* < 0·05 and 80 % power. This would be considered a small-medium effect size^(21)^.

#### Dietary intake

Dietary intake data were collected using Intake24. Intake24 is an open source, online, self-completion dietary recall system which was designed to capture food intake in population wide studies. In trials, Intake24 compared favourably with face-to-face nutritional intake data collection^(22,23)^. Participants were asked to complete four consecutive days of dietary recall. On day 1, participants were sent a link and were asked to record all food and drink and timing of consumption in the previous 24 h. The following day, a link was sent for day 2 of data collection. This process was repeated until up to 4 d of dietary intake data were recorded. Two participants accidentally completed 5 d; 370 participants completed 4 d; 11 participants completed 3 d; 6 completed 2 d; and 5 participants, 1 d.

#### Mood and sleep

Data on participants’ mood for the previous 24 h, and how well they slept the night before, were also collected before completing each day’s dietary recall. These data are not analysed here but will form the focus of a future study.

#### Food consumption variables

In line with the approach adopted by^(10)^, we extracted sixteen variables of interest from the recall data, organised by consumption event (CE), with each CE representing a time when participants consumed an item of food or drink (including water). Details of the variables extracted are presented in Table 2. The variables were of three types: those concerning consumption amounts; those concerning the intra-day patterning of CE; and those concerning inter-day variation in eating patterns. The consumption amount variables were expressed as daily averages. The consumption of different macronutrients was adjusted for total energy intake (i.e. the residual of a regression on amount of macronutrient consumption on total energy intake). In^(10)^, the inter-day variables were (unsigned) difference scores between the 2 d, since there were 2 d of food recall data for each participant in NHANES. For the present UK sample, since there were up to 5 d of recall per participant, the inter-day variables were standard deviations across days rather than difference scores.

#### Ethical standards

All procedures involving research study participants were approved by the Newcastle University Faculty of Medical Sciences Ethics Committee (ref: 24577). Informed consent was obtained from all subjects.

### Data analysis strategy

All data analyses were performed in R^(24)^.

#### UK data

Our data analysis closely followed that of Nettle and Bateson^(10)^. We first used multi-variate analyses of variance to investigate differences between food secure and food insecure adults for each of the three sets of consumption variables, using FI status as the predictor. The three sets of consumption variables were: consumption amounts (total energy intake and relative carbohydrate, protein, fat and fibre intake); intra-day patterns of eating (time of the first CE, number of CE, mean number of foods per CE, variability in time gaps between CE and variability in the amount of energy consumed at each CE) and inter-day patterns of eating (energy intake, time of the first CE, number of foods, number of CE and mean time gaps between CE). We then followed up these multi-variate analyses of variance with separate general linear models for each of the sixteen variables individually. We also tested whether FI status predicted BMI.

Despite the matched design, there were some demographic differences between our food secure and food insecure groups (see Table 1). However, in view of the sample size, the scope for including many control variables was limited. We therefore included income band and sex as controls, since we judged income to be the most critical to patterns of food consumption, and also likely to co-vary with other demographic variables such as employment status and home ownership.

#### National Health and Nutrition Examination Survey data

To compare the patterns found in the UK data with those from NHANES 2013–2014, we repeated the analyses reported by^(10)^, but with all sexes pooled. We entered sex as a predictor. The present analysis of the NHANES data also differed from that of^(10)^ in counting participants with a score of 1 on the FI questionnaire as food secure rather than insecure. This was for closer comparability with the present study. It resulted in around 6 % of NHANES respondents receiving a different FI status than in^(10)^, and no substantive difference to the results. Other control variables (age, income, education, ethnicity and presence of children in the household) and overall analytical strategy for the NHANES data were otherwise identical to^(10)^.

## Results

### Descriptive statistics

The demographic makeup of the UK sample is summarised in Table 1. Participants were, on average, in their mid-thirties, 43 % were male, with average household incomes around £17 000. Most were employed and lived in rented accommodation or with their parents. Compared with the food secure group, the food insecure participants had lower incomes, were less likely to be employed and were less likely to own their own homes. Descriptive statistics for the main food consumption variables for the UK sample and the NHANES dataset are shown in Table 2.

**Table 2.** Variables extracted from the food recalls for the UK and NHANES datasets (Mean values and standard deviations)

	Variable name	Definition	Units	UK data		NHANES 2013–2014	
				Mean	sd	Mean	sd
	Energy intake	Total energy intake per day	kcal	1759	680	2056	878
	Carbohydrate	Carbohydrate	g	218·92	95·21	247·89	112·52
Consumption amounts	Protein	Protein	g	67·22	28·2	81·96	39·48
	Fat	Fat	g	69·8	30·93	78·5	40·07
	Fibre	Fibre	g	16·48	8·61	16·9	9·54
	First CE	Time of first CE	Hours from midnight	9·3	1·9	7·9	2·3
	Number of CE	Mean number of CE per day	Number	4·07	1·08	5·47	1·63
	Mean foods per CE	Mean number of distinct foods per CE	Number	3·41	1·02	9·63	3·3
Intra-day patterns	Variability foods per CE	Intra-day standard deviation number of distinct foods per CE	Number	1·47	0·68	5·34	1·94
	Variability time gap	Intra-day standard deviation in time gap between CE	min	93·38	45·3	109·42	55·88
	Variability energy per CE	Intra-day standard deviation kcal per CE	kcal	334·23	247·19	407·38	195·52
Inter-day variability*	IDSD energy intake	Inter-day standard deviation energy intake	kcal	587	614·94	736·11	688·83
	IDSD first CE	Inter-day standard deviation time of first CE	h	1·42	1·52	1·83	2·40
	IDSD number of foods	Inter-day standard deviation number of foods	Number	0·68	0·4	4·62	3·90
	IDSD number of CE	Inter-day standard deviation number of CE	Number	0·74	0·6	1·51	1·33
	IDSD mean time gap	Inter-day standard deviation mean time gap between CE	min	47·78	34·49	68·69	74·60

CE, consumption event; IDSD, inter-day standard deviation.

* These variables represent standard deviations for the UK data, but difference scores for the NHANES data, where there are only two recall days per participant. Hence, the descriptive statistics are not directly comparable.

### Main analyses

Results of the main analyses for both the UK and NHANES datasets are summarised in Table 3.

**Table 3.** Parameter estimates for the difference between food secure and food insecure participants, UK and NHANES datasets. Food secure is the reference category (Mean values and standard errors)

	UK dataset			NHANES dataset		
	*B*	se	*P*	*B*	se	*P*
Consumption variables	MANOVA *F*(5 385) = 1·21		0·30	MANOVA *F*(54 946) = 41·80		< 0·001
Energy intake per day	–39·12	66·14	0·55	10·94	32·13	0·73
Relative carbohydrate	7·75	4·15	0·06	7·92	1·45	< 0·001
Relative protein	–3·23	1·70	0·06	–4·30	1·02	< 0·001
Relative fat	–1·38	1·50	0·36	–2·27	0·84	0·007
Relative fibre	–1·23	0·70	0·08	–1·35	0·27	< 0·001
Intra-day pattern variables	MANOVA *F*(6 378) = 3·02		< 0·001	MANOVA *F*(64 727) = 44·55		< 0·001
First CE	2·28	11·83	0·85	–0·07	0·09	0·45
Number of CE	–0·12	0·11	0·26	–0·12	0·06	0·07
Mean foods per CE	–0·28	0·10	0·01	–0·55	0·12	< 0·001
Variability foods per CE	–0·23	0·07	< 0·001	–0·39	0·07	< 0·001
Variability time gap	9·44	4·60	0·04	8·04	2·14	< 0·001
Variability energy per CE	–5·19	24·50	0·8	5·23	7·14	0·46
Inter-day variability variables	MANOVA *F*(5 364) = 1·23		0·32	MANOVA *F*(54 365) = 12·37		< 0·001
IDSD energy intake	34·07	62·30	0·58	48·11	28·75	0·08
IDSD first CE	17·27	9·26	0·06	–0·01	0·10	0·96
IDSD number of foods	–0·04	0·05	0·42	0·25	0·17	0·14
IDSD number of CE	0·04	0·04	0·41	0·09	0·09	0·13
IDSD mean time gap	6·72	3·59	0·06	5·54	3·07	0·07

CE, consumption event; IDSD, inter-day standard deviation.

#### UK dataset

There was no significant effect of FI overall for the five food consumption variables. In follow-up univariate analyses, there were marginally non-significant tendencies for food insecure respondents to consume more carbohydrate, less protein and less fibre relative to their total energy intake than their food secure counterparts did. There was no significant difference by FI status in total energy intake or relative fat consumption. There was a significant overall effect of FI on intra-day patterns of eating. Food insecure adults consumed a significantly smaller and less variable number of foods per CE and had significantly more variable time gaps between CE. However, there was no significant difference in the time of the first CE, the number of CE or the amount of energy consumed at each CE. There was no significant effect of FI on the inter-day patterns of eating, either in the multi-variate analysis of variance or in relation to any of the individual variables.

**Fig. 2.**
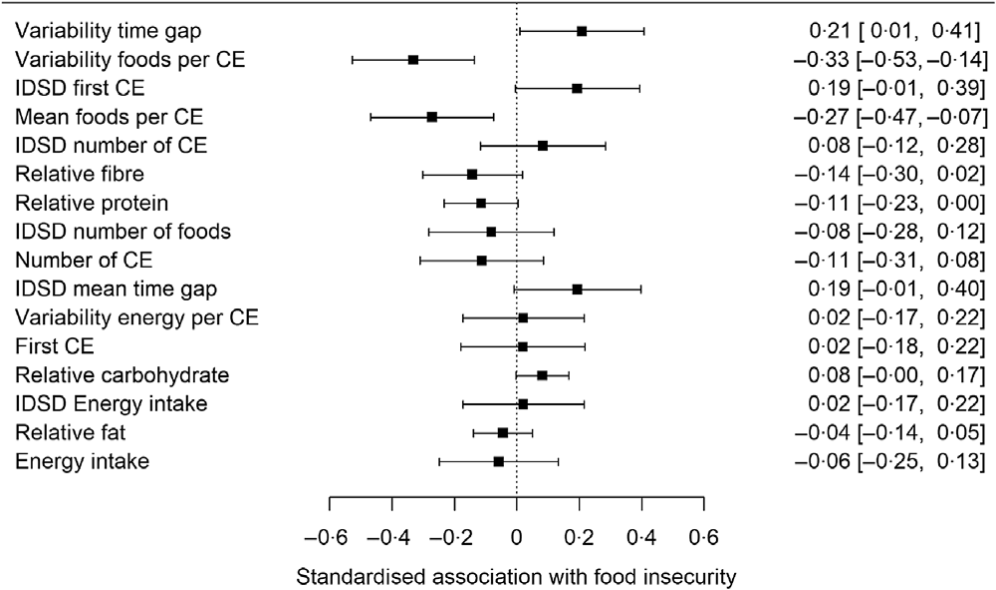
Standardised associations (plus 95 % confidence interval) of the sixteen dietary variables with food insecurity status in the UK dataset. The order in which the variables appear is for direct comparability against Fig. 1 of Nettle and Bateson^(10)^. IDSD, inter-day standard deviation.

Standardised parameter estimates, plus 95 % confidence intervals, from the univariate analyses are shown in Fig. 2. The variables are ordered from the largest to the smallest absolute parameter estimate in^(10)^, rather than the order of their size in the UK dataset.

#### National Health and Nutrition Examination Survey dataset

For the five food consumption variables, there was a significant effect of FI overall. Food insecure adults consumed significantly more carbohydrate, but less protein, fat and fibre relative to energy intake than food secure adults. Total energy intake did not however differ significantly. There was a significant effect of FI overall on intra-day patterns of eating. Food insecure adults ate a smaller and less variable number of foods at each CE and had fewer distinct foods at each CE with more variable time gaps between CE. The difference in number of CE was marginally non-significant. There was no significant difference in the time of the first CE, nor in the total amount of energy consumed at each CE. There was a significant effect overall of FI on inter-day variation in patterns of eating. However, in the follow-up analyses, none of the individual variables differed significantly by FI status.

#### Comparison of associations with food insecurity in UK and National Health and Nutrition Examination Survey datasets

**Fig. 3.**
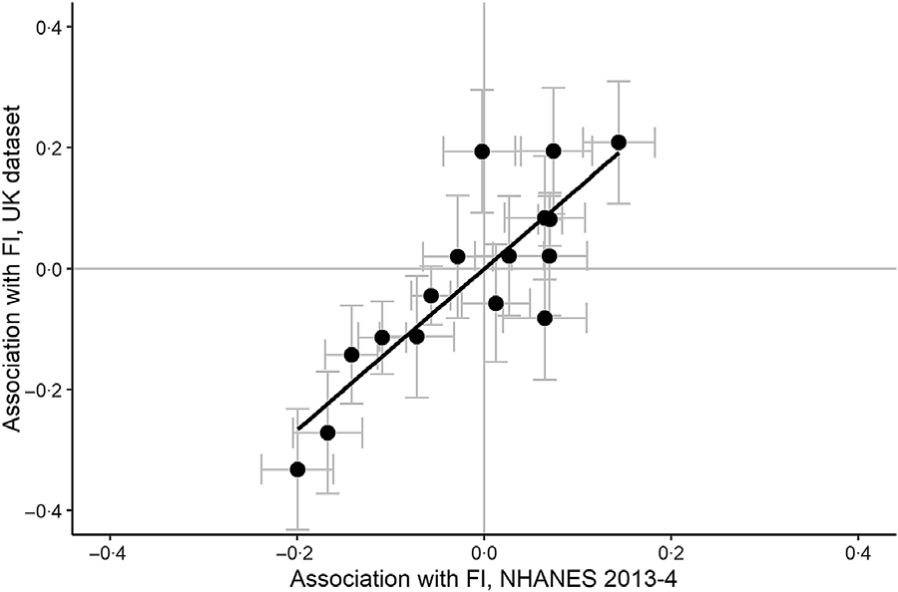
Standardised associations with food insecurity status in the NHANES 2013–2014 dataset against those in the UK dataset. Each point represents one variable. Error bars represent one standard error in the corresponding dataset.

Comparing which associations are significant in the UK and NHANES data is a poor way of assessing the similarity of patterns, since the much smaller sample from the UK means that associations are estimated with much less statistical power. Therefore, to investigate the extent to which being food insecure is associated with the same patterns of eating in our UK sample as in the NHANES sample, we compared the standardised parameter estimates related to FI status for each of the sixteen consumption variables. The pattern of associations was extremely similar for the two samples (correlation of parameter estimates between UK sample and NHANES, *r* 0·85, *P* < 0·001; see Fig. 3).

#### Association between food insecurity and BMI

There was no significant association between BMI and FI status in the UK sample, even including an expected interaction with sex in the model: B_female_ = 1·19, se 1·12, *t* = −1·06, *P* = 0·29; B_FI_ = 0·10, se 1·13, *t* = 0·08, *P* = 0·94; B_interaction_ = 0·19, se 0·91, *P* = 0·91). Thus, we did not investigate mediation of the FI–-BMI association by food consumption variables.

## Discussion

In a sample of 394 UK adults who provided a mode of four 24 h dietary recalls each, food secure and food insecure individuals did not differ in total energy intake. Food insecure participants did however have less diverse intake, as indicated by a smaller (and, concomitantly, less variable) number of distinct foods per CE. Food insecure participants also had more variable time gaps between CE than food secure participants. No other differences by FI status were significant by conventional criteria, though there were marginally non-significant trends for food insecure individuals to consume less fibre and protein, and more carbohydrate, relative to their total intake.

These findings corresponded very closely to those for the US population established using the representative NHANES 2013–2014 dataset by Nettle and Bateson^(10)^. Notably, in that study, as here, the difference in total energy intake by FI status was negligible, and the largest differences by FI status were in diversity of distinct foods, and in temporal regularity of intake. Some of the associations differed between the UK and NHANES datasets in terms of statistical significance, but this is to be expected because of the much larger NHANES sample size. The magnitude and direction of the associations were strikingly similar in the two cases. Specifically, differences in macronutrient composition – FI being associated with greater relative intake of carbohydrate and lesser of protein and fibre – were much weaker than those involving diversity and temporal regularity. This meant that they were significant in the large NHANES sample but missed criteria for significance in the smaller UK dataset.

The lack of difference in total energy intake accords with most previous studies of FI^(9,13,15,25)^, though some have found reduced energy intake^(14,26)^. This means that the higher BMI that is robustly associated with FI in women^(8)^ is apparently not driven by increased habitual energy consumption. (Note that in the present dataset, there was a null association between FI and BMI. However, our study was only modestly powered to detect such an association given the expected effect size, and the association is very well established from larger studies^(8)^.) The finding that food insecure participants had more variable time gaps between CE is potentially relevant to this puzzle. Aspects of the temporal patterning of eating have been found to co-vary with obesity in a number of studies^(10,27–30)^. Moreover, there is evidence from randomised control trials that the same number of energy content consumed on a more irregular temporal schedule has greater obesogenic potential^(31,32)^. It is possible that temporal irregularity is the factor responsible for some of the health consequences of FI.

The fact that FI was associated with more variable time gaps between food CE, here as in Nettle and Bateson, connects the human FI literature to a rich experimental tradition in birds, in which time gaps between periods of food access are made more variable and/or unpredictable^(33–38)^. Those studies too show that FI causes weight gain, generally without a concomitant increase in energy intake. In the avian case, it appears to do so by changes in digestive or metabolic efficiency^(36)^, and reducing some components of energy expenditure^(39,40)^. These possible pathways should therefore be investigated in humans too.

It is not clear why FI should increase the variability of time gaps between eating. To discover this would require richer study of which food resources participants are accessing, how and where. One obvious possibility is that there are times in the week or month when, for financial reasons, food insecure participants are skipping meals, and other times when they do not have to. However, such a pattern would show up primarily in the inter-day standard deviation of time gap between meals. The stronger association, in both datasets, is with the intra-day standard deviation in time gap. This might suggest that FI interferes with the ability to plan or choose a personal food consumption schedule, instead forcing people to rely on opportunities that present themselves at irregular moments or places.

The UK study had several limitations. First, the sample was small: the small number of participants compared with NHANES is not completely offset by the greater precision that arises from having more than two 24-h food recalls per participant. Second, we relied on an opportunity sample. Although we restricted screening to low-income participants to draw from a relatively homogenous pool, matched participants by age, and adjusted for income, unmeasured socio-economic or contextual differences between our food secure and food insecure groups may remain, and generalisability to the wider UK food insecure population is unknown. Third, our data collection took place during the coronavirus pandemic of 2020. This has a number of implications. It meant we had to assess FI with respect to the pre-pandemic period, but the dietary recalls themselves took place during the pandemic-affected period. Respondents were more likely to have been at home than usual, may have had routines disrupted and many were affected by economic hardship. These uncertainties militated against finding stable associations of dietary intake patterns with (pre-pandemic) exposure to FI. Given these uncertainties, it is, perhaps, all the more remarkable that we found significant associations between FI status and dietary intake patterns, and in particular that these would be so strikingly similar to those of the earlier study in a different population unaffected by the pandemic. This does suggest that the FI questionnaire proxies some repeatable, systematic, general differences in patterns of dietary intake.

A further limitation is the possibility of biased reporting or under-reporting in dietary recalls. For under-reporting to affect our conclusions, there would have to be differential under-reporting by FI status. This is certainly possible. Under-reporting in nutritional intake studies is often associated with social desirability, low income and high BMI^(41–45)^. Thus, it could also be associated with FI status. Under-reporting has been particularly highlighted in relation to total energy intake. Our significant findings concern other variables, such as temporal gaps, and the extent to which these are affected by under-reporting or biased reporting is not known.

### Conclusion

In a sample of UK adults, we found that the strongest dietary intake correlates of FI were lower diversity of foods, and greater variability in the time gaps between eating. Total energy intake did not differ by FI status. The findings closely mirrored those of a recent study in a US sample and suggest that the signatures of FI for dietary intake generalise across populations.

## Supplementary material

For supplementary material accompanying this paper visit http://doi.org/10.1017/S0007114521003810. click here to view supplementary material
