# Supplementary material for: Food insecurity and patterns of dietary intake in a sample of UK adults
Source: Br J Nutr. 2021 Sep 23;128(4):770–7. doi: 10.1017/S0007114521003810 (PMC9346616; doi:10.1017/S0007114521003810)
Supplement: Supplementary file 1 [file S0007114521003810sup001.docx]

Table S1. Demographic profile of phase 1 screening exercise participants, by gender and food insecurity status. Data are frequencies or mean and standard deviation (SD).

|  | **Male** | | **Female** | | **Other** |
| --- | --- | --- | --- | --- | --- |
| **Characteristic** | **Food secure** | **Food insecure** | **Food secure** | **Food insecure** | **Food**  **secure** |
| N | 500 | 98 | 764 | 134 | 4 |
| Mean age (SD) | 38 (13) | 35 (11) | 38 (13) | 34 (10) | 29 (3) |
| Employed | 339 | 59 | 525 | 82 | 2 |
| Unemployed/Furloughed/  Retired/Ill health etc. | 161 | 39 | 243 | 52 | 2 |
| Household type |  |  |  |  |  |
| Homeowner | 221 | 12 | 339 | 35 |  |
| Rent | 153 | 54 | 225 | 73 |  |
| Live with parents | 126 | 32 | 144 | 28 | 4 |
| Number of people living in the household (SD) | 2.6 (1.2) | 2.8 (1.3) | 2.8(1.1) | 3.1 (1.4) | 3.2(1.9) |
